# Outpatient ureteric stent removal following kidney transplantation

**DOI:** 10.1093/bjs/znab223

**Published:** 2021-08-26

**Authors:** F. Georgiades, A. N. S. Silva, K. Purohit, S. King, N. Torpey, K. Saeb-Parsy, G. J. Pettigrew, F. J. Rouhani

**Affiliations:** Department of Surgery, University of Cambridge, and Cambridge National Institute for Health Research Biomedical Research Centre, Cambridge, UK; Department of Surgery, University of Cambridge, and Cambridge National Institute for Health Research Biomedical Research Centre, Cambridge, UK; Department of Surgery, University of Cambridge, and Cambridge National Institute for Health Research Biomedical Research Centre, Cambridge, UK; Transplant Unit, Addenbrooke’s Hospital, Cambridge University Hospitals NHS Foundation Trust, Cambridge, UK; Transplant Unit, Addenbrooke’s Hospital, Cambridge University Hospitals NHS Foundation Trust, Cambridge, UK; Department of Surgery, University of Cambridge, and Cambridge National Institute for Health Research Biomedical Research Centre, Cambridge, UK; Department of Surgery, University of Cambridge, and Cambridge National Institute for Health Research Biomedical Research Centre, Cambridge, UK; Department of Surgery, University of Cambridge, and Cambridge National Institute for Health Research Biomedical Research Centre, Cambridge, UK

## Abstract

This article documents a single-centre experience with the use of the Isiris^TM^ single-use flexible cystoscope with integrated grasper system for the removal of ureteric stents from kidney transplant recipients in the operating theatre or in clinic. The data demonstrated that the Isiris^TM^ cystoscope can be used safely for ureteric stent removal in transplant recipients, and that an outpatient ureteric stent removal service is feasible, cost-effective, and safe in experienced hands.

## Introduction

During kidney transplantation, the Lich–Gregoir ureteroneocystostomy is routinely protected by temporary placement of a double-J ureteric stent^1^. This reduces the incidence of major urological complications, such as urine leak or transplant ureteric stenosis^2^. However, the presence of the ureteric stent is associated with an increased risk of developing urinary tract infections (UTIs) in immunocompromised transplant recipients^2^^,^^3^. The timing of stent removal has been long debated, with RCTs^4–7^ reaching conflicting conclusions, depending on the outcome measured. Early removal of ureteric stents (within 7 days of implantation) appears to decrease the incidence of UTIs, but may increase the incidence of major urological complications^3^^,^^8^, and consequently, many transplant units aim for removal at 6 weeks after transplantation.

In the UK, this usually necessitates recipients being admitted for a day-case stent removal procedure in the operating theatre under local anaesthesia (LA). Performing ureteric stent removals in resource-intensive operating theatres has high associated costs. Furthermore, growing healthcare service demands, particularly the increasing pressure on elective theatre availability^9^, can result in frequent cancellation of operations with consequent procedural delays and poor patient experience. Therefore, it is timely to consider whether transplant stent removal could be performed on an outpatient basis. In this regard, a single-use disposable flexible cystoscope with an integrated grasper system (Isiris^TM^; Coloplast, Humlebaek, Denmark) has been developed. This system has been used in the outpatient setting for removal of native ureteric stents^10^^,^^11^, but similar outpatient management of ureteric stents in the transplant patient population has not been described to date.

The aim of this study was to describe the feasibility, safety, and costs associated with a dedicated flexible cystoscope for the removal of ureteric stents from transplant recipients in the outpatient and operating theatre departments.

## Methods

This was a single-centre retrospective study of kidney transplant recipients who underwent stent removal in an operating theatre and outpatient setting between May 2017 and March 2020. Details of the study design, methods employed, immunosuppression protocols, and statistical analysis are described in detail in *Appendix S1*.

## Results

Some 533 transplant recipients attended for stent removal under LA using a dedicated flexible cystoscope (Isiris^TM^). The theatre cohort consisted of 221 patients, of whom 152 (68.8 per cent) were male, and the outpatient cohort consisted of 312 patients, of whom 198 (63.5 per cent) were male (*Table S1*). The mean age of recipients was 49.1 (range 17–79) years in the theatre cohort and 50.7 (18–80) years in clinic cohort (*P* = 0.196). The majority of patients, 192 (86.9 per cent) in the theatre cohort and 280 (89.7 per cent) in the clinic cohort, had the stent removed after an isolated kidney transplant. The remaining transplant recipients received a kidney as part of a multiple-organ transplant (*Table S1*). The majority of transplant recipients in this study (440) received organs from deceased donors (*Table S1*). For some patients, two stents were explanted, owing to dual transplants (5) or kidneys with double ureters (6). In one patient, a native ureteric stent was removed alongside the transplanted ureteric stent in clinic.

In total, 227 ureteric stents were removed from the 221 transplant recipients in theatres and 313 stents from 312 recipients in the outpatient clinic. Four patients (1.3 per cent) in the clinic cohort did not tolerate the procedure under LA, and the stents were subsequently removed in theatres under general anaesthesia (GA). As a result of needing GA, in contrast to the rest of the cohort, they were excluded from subsequent analysis. Earlier removal of stents was achieved in clinic compared with theatre (47.7 *versus* 53.4 days after transplant; *P* = 0.001) (*Table 1*), which was closer to the unit’s target of 42 days after transplantation.

**Table 1:** Stent removal in kidney transplant recipients using Isiris^TM^ cystoscope

	Theatre (*n* = 221)	Clinic (*n* = 312)	Total (*n* = 533)	*P* ^†^
**Time since implantation (days)** *	53.4(18.5) (11–128)	47.7(14.5) (16–202)	50.1(16.5) (11–202)	<0.001^‡^
**Intraprocedural complications**				0.858
None	206 (93.2)	293 (93.9)	499 (93.2)	
Clavien–Dindo grade I–III	15 (6.8)	19 (6.1)	34 (6.3)	
**Follow-up at 2 weeks after stent removal**				
Hospital admission	27 (12.2)	20 (6.5)	47 (8.9)	0.029
Unexpected admission from clinic	17 (7.7)	18 (5.8)	35 (6.6)	0.478
Emergency admission	10 (4.5)	2 (0.6)	12 (2.3)	0.005
Complications (Clavien-Dindo classification)				0.403^§^
None	194 (87.7)	288 (93.5)	482 (91.1)	
I	5 (2.3)	2 (0.6)	7 (1.3)	
II	10 (4.5)	5 (1.6)	15 (2.8)	
IIIa	10 (4.5)	8 (2.6)	18 (3.4)	
IIIb	1 (0.5)	4 (1.3)	5 (0.9)	
IVa	1 (0.5)	1 (0.3)	2 (0.4)	

Values in parentheses are percentages unless indicated otherwise;

*values are mean (s.d.) (range).

† Fisher’s exact test, except.

‡ Mann–Whitney *U* test and ^§^χ^2^ test.

During stent removal, there were complications in 15 (6.8 per cent) and in 19 (6.1 per cent) patients in the theatre and clinic cohorts respectively (*Table S2*). There was no difference between the two settings in renal graft function after the procedure (*Fig. S1*).

Most patients in both groups received ciprofloxacin as prophylaxis. In patients with an allergy to ciprofloxacin, one of co-amoxiclav, gentamicin, nitrofurantoin or vancomycin was used instead (*Table S3*). Only two patients (0.9 per cent) in the theatre cohort and two (0.8 per cent) in the clinic cohort did not receive any antibiotic prophylaxis (*P* = 0.262).

Microbiology cultures from the retrieved stents were positive in 96 (43.4 per cent) and 148 (48.1 per cent) patients in the theatre and clinic cohorts respectively (*P* = 0.331). Of these, antibiotic-resistant microorganisms were isolated in one and 11 patients respectively (*P* = 0.031). However, some were present before the procedure; in all, new isolates were grown in one patient in the theatre and eight in the clinic cohort (*P* = 0.087) (*Table S3*).

Admissions within 2 weeks of stent removal were scrutinized to capture potential complications following cystoscopy (*Table 1*). In total, 47 transplant recipients (8.9 per cent of total) were admitted from both settings. Twenty were in the clinic cohort (6.5 per cent) and none of these complications were related to the procedure itself (*Table S4*). In comparison, there were 27 admissions in the theatre cohort (12.2 per cent) (*P* = 0.029), of which three (1.4 per cent) were for UTIs after cystoscopy. There was no significant difference in readmission rates of patients who had kidney transplants in this unit over the two time periods (8.8 readmissions per month during the time period of data capture for the theatre cohort and 9.8 readmissions per month for the clinic cohort; *P* = 0.410). Of the readmissions, 10 (4.5 per cent) in the theatre cohort and two (0.6 per cent) in the clinic cohort presented to the emergency department (*P* = 0.005). Most readmissions from the transplant clinic were for investigation of graft dysfunction or transplant-related complications (*Table S4*). For those admitted, the mean duration of hospital stay was 6.8 days in the theatre cohort and 7.8 days in the clinic cohort (*P* = 0.632). There were no deaths within 30 days of cystoscopy in either setting.

The calculated beneficial difference in cost was €909 per patient for stent removal in clinic instead of theatres (*Appendix S2* and *Table S5*). Twenty-five patients in the clinic cohort were given Patient Satisfaction Questionnaires (*Appendix S3*) and rated the service with a mean score of 9.68 of 10 (*Appendix S2* and *Table S6*).

## Discussion

Outpatient clinic cystoscopy and associated procedures are well established in general urology practice^11^, but not for the transplant population, particularly in the UK. This study described a single-centre experience with moving stent removal after kidney transplant from an operating theatre environment to an outpatient clinic setting. The study demonstrated that the outpatient clinic setting was feasible and safe, with stent removals closer to a target of 42 days after transplantation.

The rates of procedural complications, incidence of UTIs, and isolation of micro-organisms, including antibiotic-resistant strains, were not significantly different between the theatre and clinic cohorts. However, readmission rates within 2 weeks of the procedure were higher among the theatre cohort. These results show that stent removal in a dedicated outpatient clinic is safe and does not result in greater rates of complications or admissions.

Moving the procedure from the theatres to outpatients may also result in cost savings. Analysis carried out by the hospital finance department showed a surplus of £781 per procedure, which could then be reinvested back into the service. However, this saving will be partially offset by procedures in which additional scopes are required or that need rebooking under GA. Additional benefits of establishing an outpatient service include freeing up theatre capacity, which will in turn benefit waiting lists and opportunities for dedicated specialist nurse-led clinics^12^^,^^13^.

This work has some limitations, namely that it was a retrospective study and involved sequential periods of analysis, during which there would have been a learning curve and growing expertise in the use of the Isiris™ system. The focus was also only on cystoscopic stent removals, whereas in recent years alternative methods have been described in transplant recipients; these include suturing the stent on to the urethral catheter, for both to be removed at the same time^14^^,^^15^, and, more recently, the use of magnetic stents (Black-Star^®^; Urotech (Rohrdorf OT Achenmühle, Germany)) and their magnetic removal^16^. These offer less invasive approaches, but further studies are needed to evaluate their safety and cost-effectiveness compared with cystoscopic removal, which remains the standard. In addition, the widespread use of disposable cystoscopes may have an environmental impact which will require detailed analysis in future studies.

The Isiris^TM^ single-use integrated grasper system can be used in the kidney transplant population for ureteric stent removal under LA in both theatre and outpatient settings. Intraprocedural complication, microbiological, and biochemical outcomes were similar in outpatients *versus* theatre. However, when used in the outpatient setting, the procedure is cheaper, can offer shorter stent removal intervals, lower readmission rates, and is highly rated by patients. A dedicated outpatient ureteric stent removal service for kidney transplant recipients appears to be feasible, cost-effective, and safe.

## Supplementary Material

znab223_Supplementary_Data
