# Supplementary material for: Outpatient ureteric stent removal following kidney transplantation
Source: Br J Surg. 2021 Aug 26;109(2):152–4. doi: 10.1093/bjs/znab223 (PMC10364773; doi:10.1093/bjs/znab223)
Supplement: znab223_Supplementary_Data [file znab223_supplementary_data.zip › Patient Satisfaction Questionnaire.pdf]

Transplant Stent Clinic Patient Satisfaction Questionnaire

This questionnaire is about your **most recent** experience of the **removal of ureteric stent/s** at Cambridge University Hospitals

What you tell us is confidential and taking part is voluntary.

WHO SHOULD COMPLETE THE QUESTIONNAIRE?

The questions should ideally be answered by the patient If that person needs help to complete the questionnaire, the answers should be given from their point of view – not the point of view of the person who is helping.

WHAT TO DO

Put a cross ☒ clearly inside one box using a black or blue pen.

If you make a mistake, just fill in the box ■ and put a cross ☒ in the correct box.

If you cannot answer a question, or do not want to answer it, just leave it blank and go to the next question.

NEED MORE HELP?

For help completing the questionnaire, please contact the Renal Transplant Co-ordinators on **01223 348031** or **01223 274637** or speak to the member of staff that has provided this form.

If you have concerns about the care you have received, please speak to a member of staff who can advise you about the PALS service.

APPOINTMENT

1. Was your appointment date changed by the hospital?

1 ☐ No

2 ☐ Yes, once

3 ☐ Yes, 2 or 3 times

4 ☐ Yes, 4 times or more

2. At any stage, did you feel that you had to wait a long time to be seen?

1 ☐ Yes, definitely

2 ☐ Yes, to some extent

3 ☐ No

THE HOSPITAL

3. In your opinion, how clean was the hospital room that **you** were in?

1 ☐ Very clean

2 ☐ Fairly clean

3 ☐ Not very clean

4 ☐ Not at all clean

HEALTHCARE STAFF

4. When you had important questions to ask, did you get answers that you could understand?

1 ☐ Yes, always

2 ☐ Yes, sometimes

3 ☐ No

5. Did you have confidence and trust in the healthcare staff treating you?

1 ☐ Yes, always

2 ☐ Yes, sometimes

3 ☐ No

YOUR CARE & TREATMENT

6. Were you involved as much as you wanted to be in decisions about your care and treatment?

1 ☐ Yes, definitely

2 ☐ Yes, to some extent

3 ☐ No

7. Were you given enough privacy when discussing your condition or treatment?

1 ☐ Yes, always

2 ☐ Yes, sometimes

3 ☐ No

8. Were you given enough privacy when being examined or treated?

1 ☐ Yes, always

2 ☐ Yes, sometimes

3 ☐ No

9. Were you ever in any pain?

1 ☐ Yes

2 ☐ No

10. Do you think the hospital staff did everything they could to help control your pain?

1 ☐ Yes, definitely

2 ☐ Yes, to some extent

3 ☐ No

11. If you needed attention, were you able to get a member of staff to help you **within a reasonable time**?

1 ☐ Yes, always

2 ☐ Yes, sometimes

3 ☐ No

4 ☐ I did not want / need this

OPERATIONS & PROCEDURES

12. Beforehand, did a member of staff answer your questions about the operation or procedure in a way you could understand?

1 ☐ Yes, completely

2 ☐ Yes, to some extent

3 ☐ No

4 ☐ I did not have any questions

13. Beforehand, were you told how you could expect to feel after you had the operation or procedure?

1 ☐ Yes, completely

2 ☐ Yes, to some extent

3 ☐ No

14. I would rather have had this procedure in the operating room?

1 ☐ Yes, definitely

2 ☐ I'm not sure

3 ☐ No

LEAVING HOSPITAL

15. When you left hospital, did you know what would happen next with your care?

1 ☐ Yes, definitely

2 ☐ Yes, to some extent

3 ☐ No

16. Did a member of staff tell you about any danger signs you should watch out for after you went home and who to contact should this happen?

1 ☐ Yes, completely

2 ☐ Yes, to some extent

3 ☐ No

OVERALL

17. If I were to have this procedure again in the future, I would rather have a formal admission to hospital?

1 ☐ Yes, always

2 ☐ Yes, sometimes

3 ☐ No

18. Overall, did you feel you were treated with respect and dignity while you were in the hospital?

1 ☐ Yes, always

2 ☐ Yes, sometimes

3 ☐ No

19. Overall... (**Please circle a number**)

I had a very poor experience — I had a very good experience

0 1 2 3 4 5 6 7 8 9 10

Cambridge University Hospitals NHS Foundation Trust will hold your information securely in accordance with the Data Protection Act (1998).

We may share information you provide with our services as part of our ongoing commitment to improving the quality of the services we deliver.

Please tick here if you are NOT happy for us to use your feedback in this way. ☐

Please tick here if you are NOT happy for your feedback to be used anonymously on service information leaflets and webpages ☐

OTHER COMMENTS

If there is anything else you would like to tell us about your experiences in the hospital, please do so here.

Was there anything particularly good about your hospital care?

Was there anything that could be improved?

Any other comments?

THANK YOU VERY MUCH FOR YOUR HELP

If you are happy for our Transplant Co-ordinators to contact you about your responses, please enter your contact details below:

Name:
Contact telephone number:
