# Supplementary material for: Outpatient ureteric stent removal following kidney transplantation
Source: Br J Surg. 2021 Aug 26;109(2):152–4. doi: 10.1093/bjs/znab223 (PMC10364773; doi:10.1093/bjs/znab223)
Supplement: znab223_Supplementary_Data [file znab223_supplementary_data.zip › Supplementary_information.docx]

# **Supplementary information**

## **Supplementary Appendix 1- Detailed Materials and Methods**

## **Study design and stent removal using the disposable flexible cystoscope – Isiris^TM^**

From the introduction of the Isiris^TM^ scope by Coloplast (Humlebaek, Denmark) in clinical practice in our unit in May 2017, data was collected retrospectively for all transplant recipients that had their transplant ureteric stents removed, from 2nd May 2017 until 5th March 2020. This included two cohorts of patients: those who attended theatre for the procedure (May 2017 to August 2018) and those who attended the newly set up transplant ureteric stent clinic (August 2018 to March 2020) at our centre. Patients who were part of an ongoing separate early stent removal trial (removed <10 days post-transplant) were excluded from this study as were those patients who had their stent removed under general anesthesia (GA).

For all patients, stents were removed during a single patient encounter (e.g. a clinic visit or day attendance for theatre), after obtaining written consent, using the single-use disposable flexible cystoscope (Isiris^TM^) under local anesthesia (LA). Antibiotic prophylaxis was routinely given as a single dose within 30 minutes prior to the procedure. A single medically trained operator (a surgical specialist registrar or senior clinical fellow level with more than 5 years of clinical practice) performed the procedure with an assistant (nurse or healthcare assistant), who would help maintain sterility by connecting the portable LCD monitor and the irrigation fluid to the cystoscope. Briefly, under sterile conditions, 11ml of 2% lidocaine containing lubricant gel (Optilube^TM^ by Optimum Medical, Leeds, UK) was administered intra-urethrally just before the introduction of the 16.5Fr scope using sodium chloride 0.9% as irrigation fluid to aid in passage of the Isiris^TM^ integrated grasper system to the urinary bladder. The retrieved ureteric stents were sent for microbiological culture. For each patient, documentation also included intra-procedural complications and whether encrustation of the stent was observed at the time of retrieval. Complications were reported using the revised Clavien-Dindo classification^1^.

## **Cambridge kidney transplant protocol**

The Cambridge Transplant Unit’s kidney transplant immunosuppression protocol for standard immunological risk transplant recipients uses Basiliximab 20mg IV (day 0 and day 3) and Methyl-prednisolone 10mg/Kg IV (intra-operatively) for induction, followed by a reducing regimen of prednisolone starting at 20mg, Mycophenolate Mofetil (MMF) 750 mg PO BD and Tacrolimus MR 0.15 mg/kg [dose adjusted to achieve a target serum level of 8 μg/L (range 7–10 μg/L)]. Recipients classed as high immunological risk, those receiving a pancreas (SPK) or multi-visceral transplant (MVTx) or those with high BMI or at risk of diabetes, were planned for a steroid free regimen with Alemtuzumab 30 mg SC induction followed by Tacrolimus MR 0.15 mg/Kg and a reduced dose of MMF at 500 mg PO BD.

The urinary catheter inserted at the time of transplant is removed on post-operative day 5. All patients are started on regular co-trimoxazole for 6 months as part of our hospital’s Pneumocystis and fungal infection prophylaxis protocol. Following discharge, the patients are reviewed in clinic twice a week for the first 3 weeks and weekly thereafter until stent removal in theatre/clinic. At the time of stent removal, the recipients induced with basiliximab would typically be on 5 mg prednisolone PO OD, MMF and tacrolimus and those on steroid free regimens with alemtuzumab induction, on MMF and tacrolimus for maintenance immunosuppression.

## **Microbiology related data**

Data from individual stent cultures that were processed following removal of stents were collected for each patient. Microbiological results were categorized into contaminants (microorganisms usually found as part of skin flora) or urinary tract pathogens (microorganisms known to cause urinary tract infections (UTI) e.g. Gram-negative bacteria). Any resistant strains were also identified and classified.

## **Hospital re-admissions and emergency department attendance**

Hospital readmission rates were determined by averaging monthly readmissions for kidney transplant recipients during the corresponding time period for each setting. Emergency department attendances and hospital admissions to our unit or regional hospitals within 14 days of the procedure were recorded for all patients during follow-up clinic visits and from the transplant coordinator team.

## **Impact of location of stent removal on graft function assessment**

For all patients, serum creatinine levels (Cr) were measured at routine clinic appointments before and after the procedure. A proportion of patients in both settings were identified to have had a Cr check more than 7 days post procedure; therefore, data were dichotomized to subgroups for each setting, ≤7 days and >7 days interval creatinine check. To compare renal function before and after stent removal between the two subgroups, changes in the serum creatinine difference (Δ_Cr_) from the same individual were calculated (Δ_Cr_= Cr_Pre_-Cr_Post_).

## **Cost analysis**

The net income that a procedure generates for a hospital trust in the National Health Service is calculated using a renumeration tariff offset by the cost, both of which are standardized per procedure. The gross income per procedure is based on the services provided for each setting (theatre vs outpatients) and was calculated per patient based on current remuneration tariffs of the National Health Service (NHS). Following each patient encounter, the clinical information (the procedure and the setting: theatre or outpatient clinic) was coded to represent grouped interventions that use a pre-specified amount of NHS resources, and therefore determine the final tariff received by the NHS trusts^2^. The standardized cost of each procedure is similarly calculated by each hospital including staffing, consumables, theatre/clinic space use, intervention and investigations. Therefore, each procedure generates a balance for the hospital, whether a profit or a loss that is published publicly every year^3^. The cost associated with clinical staff employment was calculated from publicly available standardized pay circulars used in practice across the UK^4^. Theatre running costs were calculated by the finance department of the hospital using local data on capital costs using the Patient-Level Information and Costing Systems (PLICS)^5^. Conversion rates between Great Britain Pound (GBP; £) and United States Dollar (USD; $) were set at the current exchange rate of £1=€1.163=$1.336 (December 2020).

## **Patient satisfaction questionnaire**

A patient satisfaction questionnaire (PSQ) was constructed in line with guidance from the literature^6^ and existing local questionnaires, including additional questions specific to the stent clinic setting (Supplementary file 2). These were distributed to 25 consecutive transplant recipients of both genders after their follow-up clinic appointments to evaluate the quality of care they have received and to provide feedback of their experience of the stent removal clinic.

## **Statistical analysis**

Statistical analyses of the means and differences were performed using IBM SPSS® Software (Version 24, IBM Corp., New York, USA) and GraphPad Prism (Version 7.04, GraphPad Software Inc, San Diego, USA). Statistical significance was set at p-value≤0.05. For demographics comparisons Fisher’s exact test and chi-squared were used. To compare means, two-tailed unpaired t-tests were used except for the mean Cr pre- and post-removal where a two-tailed paired t-test was used for each setting. Fisher’s exact test and chi-squared test were used for the analysis of complications and the microbiology data. The study described here underwent institutional approval locally as a clinical audit.

**References for additional materials and methods**

1 Dindo D, Demartines N, Clavien P-A. Classification of surgical complications: a new proposal with evaluation in a cohort of 6336 patients and results of a survey. *Ann Surg* [Internet]. 2004 Aug; **240**: 205–213. Available from: http://www.ncbi.nlm.nih.gov/pubmed/15273542

2 Mahbubani K, Georgiades F, Goh EL, Chidambaram S, Sivakumaran P, Rawson T, *et al.* Clinician-directed improvement in the accuracy of hospital clinical coding. *Futur Healthc*. 2018; **5**: 47–51.

3 NHS Improvement. Developing the national tariff [Internet]. 2020 [cited 2020 Dec 8]. Available from: https://improvement.nhs.uk/resources/developing-the-national-tariff/

4 NHS. NHS Employers: Pay, pensions and reward [Internet]. 2020 [cited 2020 Dec 9]. Available from: https://www.nhsemployers.org/pay-pensions-and-reward

5 HFMA. Patient-Level Information and Costing Systems (PLICS) [Internet]. [cited 2020 Dec 21]. Available from: https://www.hfma.org.uk/our-networks/healthcare-costing-for-value-institute/what-is-plics

6 Lees C. Measuring the patient experience. *Nurse Res*. 2011; **19**: 25–28.

**Supplementary Appendix 2 – Additional Results**

## **Microbiology related outcomes**

Microbiology cultures from the retrieved stents were positive in 96 (43.4%) and 148 (48.1%) transplant recipients in the theatre and clinic cohort, respectively (p-value=0.331) (Table S3). Cultures from the theatre cohort grew a urinary tract pathogen in 39.6% (38 out of 96) and contaminant organisms in the rest. In contrast, cultures from the clinic cohort grew a urinary tract pathogen in 53.3% (79 out of 148) with the rest as contaminant organisms (Table S3). Isolation of antibiotic resistant strains of microorganisms was noted in 11 patients in clinic and in 1 in the theatre cohort (p-value=0.031) (Table S3). However sub-group analysis of the antibiotic resistant strains isolated from stent cultures revealed no significant difference between the organisms isolated in the two settings (p-value=0.25) since in some patients the same pathogens were pre-existing, in urine cultures prior to the stent removal (3 cases in clinic cohort and none in theatre cohort). Urine culture was not collected prior to the stent removal in 2 patients (22.2%) and in 4 (44.4%) patients the urine cultures prior to stent removal did not grow the same pathogen.

## **Post-procedure graft function**

There was no difference in serum creatinine levels observed in both settings pre and post procedure. (Supplementary Figure 1). Additionally, no statistically significant difference was observed when the ΔCr of those within the subgroups of each setting was analyzed (creatinine check ≤7 days Vs creatinine checked >7 days post stent removal) (p-value= 0.263 for clinic and 0.672 for theatre; Supplementary Figure 1).

## **Cost analysis**

The cost of scope and associated LCD screen purchase was the same between the two settings. Total expenses per patient in the theatre setting in our hospital were £1,242 ($1660) in comparison to £461 ($616) in outpatients. This accounts for a total difference of £781 ($1044) per patient, if a patient’s stent is removed in the clinic setting instead of theatres (Table S5).

## **Patient satisfaction questionnaire**

To evaluate patient satisfaction with the stent clinic service, we sought feedback from transplant recipients using our designed PSQ. 25 consecutive patients in the stent clinic were asked to fill in the questionnaire provided following their appointment, all of whom agreed. The overall response was positive with twenty-four patients (96%) reporting no pain during the procedure and found the experience of having the stent removed in clinic acceptable. Overall satisfaction was rated 9.68 (± SD 0.85) on a scale of 1 to 10 (range 6 – 10) (Table S6).

## **Major Urological Complications during study period**

No urinary leak or fistulae were observed in both settings. Ureteric stenosis was identified in n=10 patients from the clinic cohort and n=5 from the theatre cohort, during the whole study period (p-value=0.603). From these n=3 from clinic and n=1 from theatre were identified within 2 weeks from stent removal, as they required a hospital admission (Table S4).

## **Appendix 3 – Supplementary tables**

| Supplementary Table 1: Demographics of transplant recipients that required stent removal | | | | | |  |
| --- | --- | --- | --- | --- | --- | --- |
|  | **Theatre** | | **Clinic** | | **Total** | **P-value** |
|  | **Number of patients (n)** | **% of cohort** | **Number of patients (n)** | **% of cohort** | **(n)** |  |
| Gender |  |  |  |  |  | 0.228^†^ |
| Male | 152 | 68.8 | 198 | 63.5 | 350 |  |
| Female | 69 | 31.2 | 114 | 36.5 | 183 |  |
| Total | 221 |  | 312 |  | 533 |  |
| Type of Transplant |  |  |  |  |  | 0.487^‡^ |
| Kidney | 192 | 86.9 | 280 | 89.7 | 472 |  |
| SPK | 23 | 10.4 | 28 | 9.0 | 51 |  |
| SLK | 3 | 1.4 | 3 | 1.0 | 6 |  |
| MVTx + Kidney | 3 | 1.4 | 1 | 0.3 | 4 |  |
| Type of Donor |  |  |  |  |  | 0.250^‡^ |
| DCD | 92 | 41.6 | 148 | 47.4 | 240 |  |
| DBD | 92 | 41.6 | 108 | 34.6 | 200 |  |
| LD | 37 | 16.8 | 56 | 17.9 | 93 |  |
| Induction Immunosuppression |  |  |  |  |  | 0.687^†^ |
| Basiliximab | 193 | 87.3 | 276 | 88.5 | 469 |  |
| Alemtuzumab (Steroid-free) | 28 | 12.7 | 36 | 11.5 | 64 |  |
|  |  |  |  |  |  |  |
|  | **Mean(±SD)** | **Range** | **Mean(±SD)** | **Range** | **P-value** | |
| Age (years) | 49.1 (±13.5) | 17 – 79 | 50.7 (±14.2) | 18 - 80 | 0.196* | |
| Days since implantation | 53.4 (±18.5) | 11 – 128 | 47.7 (±14.5) | 16 - 202 | <0.001* | |
| SPK: simultaneous pancreas and kidney; SLK: simultaneous liver and kidney; MVTx: Multi-visceral transplant; DCD: Donation after circulatory death; DBD: Donation after brain death; LD: Living donation; SD: Standard Deviation.†: Fisher’s exact test; ‡: Chi-squared; *: unpaired t-test. | | | | | | |

| Supplementary Table 2: Stent encrustation and intra-procedure related complications. | | | | |  |
| --- | --- | --- | --- | --- | --- |
|  | **Theatre** | | **Clinic** | | **p-value** |
|  | **Number of patients (n)** | **%** | **Number of patients (n)** | **%** |  |
| Stent Encrustation | 3 | 1.4 | 10 | 3.5 | 0.170^†^ |
| Intra-procedural Complications |  |  |  |  | 0.858^‡^ |
| None | 206 | 93.2 | 293 | 93.9 |  |
| Mild bleeding | 6 | 2.7 | 9 | 2.9 |  |
| Failed retrieval - need for GA | - | - | 4 | 1.3 |  |
| Failed retrieval attempt – need for other scope in same setting | 4 | 1.8 | 2 | 0.6 |  |
| Grasper jaw malfunction | 3 | 1.4 | 1 | 0.3 |  |
| Deflection control malfunction | 1 | 0.5 | 1 | 0.3 |  |
| Difficult passage of cystoscope – pre-existing urethral stricture | 2 | 0.9 | 1 | 0.3 |  |
| Bleeding with clot formation | 1 | 0.5 | - | - |  |
| Pain requiring oral analgesia | 1 | 0.5 | - | - |  |
| Sutured stent | 1 | 0.5 | 1 | 0.3 |  |
| Resistance during stent retrieval | - | - | 1 | 0.3 |  |
| Stiff stent | - | - | 1 | 0.3 |  |
| GA: General Anaesthesia; .†: Fisher’s exact test; ‡: Chi-squared. | | | | | |

| Supplementary Table 3: Microbiology data | | | | |  |
| --- | --- | --- | --- | --- | --- |
|  | **Theatre** | | **Clinic** | |  |
|  | **Number of patients (n)** | **%** | **Number of patients (n)** | **%** | **p-value** |
| Antibiotic prophylaxis |  |  |  |  | 0.262^‡^ |
| Ciprofloxacin | 204 | 92.3 | 295 | 95.7 |  |
| Co-amoxiclav | 7 | 3.2 | 3 | 1.0 |  |
| Other | 8 | 3.6 | 8 | 2.6 |  |
| None | 2 | 0.9 | 2 | 0.7 |  |
|  |  |  |  |  |  |
| Stent Culture Growth |  |  |  |  | 0.331^†^ |
| Yes | **96** | **43.4** | **148** | **48.1** |  |
| Likely Contaminant^1^ | 58 |  | 69 |  |  |
| Urinary tract pathogen^2^ | 38 |  | 79 |  |  |
| No | **125** | **56.6** | **160** | **51.9** |  |
|  |  |  |  |  |  |
| Growth Details |  |  |  |  | 0.512^†^ |
| Monomicrobial Growth Isolates (>15 cfu) | **49** | **51.4** | **68** | **45.9** |  |
| *S.epidermidis^1^* | 23 |  | 17 |  |  |
| *E.Coli^2^* | 2 |  | 9 |  |  |
| *E.faecalis^2^* | 3 |  | 9 |  |  |
| Candida species^2^ | 3 |  | 5 |  |  |
| *E.faecium^2^* | 3 |  | 6 |  |  |
| *Proteus species^2^* | 1 |  | 3 |  |  |
| *C.Freundii^2^* | 1 |  | 2 |  |  |
| *S.haemolyticus^1^* | 3 |  | 1 |  |  |
| *E.cloacae^2^* | - |  | 4 |  |  |
| *K.pneumoniae^2^* | - |  | 3 |  |  |
| *P.aeruginosa^2^* | - |  | 2 |  |  |
| *S.aureus^1^* | - |  | 1 |  |  |
| Lactobacillus species^2^ | - |  | 1 |  |  |
| Polymicrobial Growth Isolates (>15 cfu) | **47** | **49.0** | **80** | **54.1** |  |
| Mixed skin flora^1^ | 20 |  | 39 |  |  |
| Staphylococcus spp^1^ containing | 24 |  | 25 |  |  |
| *E.Coli^2^* containing | 7 |  | 17 |  |  |
| *Enteroccoci* spp.^2^ containing | 10 |  | 15 |  |  |
| *Candida* spp*.^2^* containing | 7 |  | 10 |  |  |
| *Corynebacterium* spp.^1^ containing | 5 |  | 3 |  |  |
| Mixed Gram -ve flora | 4 |  | 3 |  |  |
| Antibiotic resistant isolates | **1** |  | **11 (8*)** |  | **0.031^†^ (0.087)** |
| Extended spectrum β-lactamase | - |  | 9 (6*) |  |  |
| Vancomycin-resistant Enterococci | 1 |  | 2 |  |  |
| .† Fisher’s exact test; ‡ Chi-squared; ^1^Likely contaminant organisms; ^2^Urinary tract pathogens; *new isolates were found in 1 theatre and 8 clinic cohort patients p=0.087; cfu: colony forming units | | | | | |

| Supplementary Table 4: Follow-up post stent removal | | |  | | |
| --- | --- | --- | --- | --- | --- |
|  | **Theatre** | | **Clinic** | | **P-value** |
|  | **Number of patients (n)** | **%** | **Number of patients (n)** | **%** |  |
| Hospital Admission within 2 weeks following cystoscopy | 27 | 12.2 | 20 | 6.5 | **0.029^†^** |
| UTI following cystoscopy | 3 |  | - |  |  |
| Renal transplant biopsy | 3 |  | 3 |  |  |
| Peri-nephric collection | 3 |  | 1 |  |  |
| Diarrhoea with AKI | 3 |  | - |  |  |
| Lymphocele | 2 |  | 2 |  |  |
| Acute rejection | 2 |  | 2 |  |  |
| Ureteric stenosis - reconstruction | 1 |  | 3 |  |  |
| Haemodialysis | 1 |  | 1 |  |  |
| PTLD | 1 |  | - |  |  |
| Intra-abdominal collection | - |  | 1 |  |  |
| AKI – spontaneous resolution | 1 |  | - |  |  |
| MRSA bacteraemia – line related | 1 |  | - |  |  |
| Renal artery stenosis – IR angioplasty | 1 |  | - |  |  |
| Community-acquired pneumonia | 1 |  | - |  |  |
| Small bowel obstruction – conservative | 1 |  | - |  |  |
| Urinary retention – failed TWOC | 1 |  | - |  |  |
| A-V fistula ligation | 1 |  | - |  |  |
| A-V Aneurysm – IR intervention | - |  | 1 |  |  |
| CNI toxicity | 1 |  | 2 |  |  |
| Catheter-associated UTI | - |  | 1 |  |  |
| Fall – Tibia & Fibula fracture - ORIF | - |  | 1 |  |  |
| Pancytopenia | - |  | 1 |  |  |
| MRSA wound infection | - |  | 1 |  |  |
| Unexpected Admission from clinic | 17 | 7.7 | 18 | 5.8 | 0.478**^†^** |
| Emergency admission | 10 | 4.5 | 2 | 0.6 | **0.005^†^** |
|  | **Mean(±SD)** | **Range** | **Mean(±SD)** | **Range** |  |
| Length of stay (days) | 6.8 (±6.8) | 1-32 | 7.8 (±6.7) | 1-27 | 0.632* |
|  |  |  |  |  |  |
| AKI: Acute kidney injury; A-V: Arterio-venous; CNI: calcineurin inhibitor; IR: interventional radiology; MRSA: Methicillin resistant staphylococcus aureus; ORIF: Open reduction internal fixation; UTI: urinary tract infection; PTLD: Post-transplant lymphoproliferative disorder; TWOC: Trial without catheter; SD: Standard Deviation. .†: Fisher’s exact test; *: unpaired t-test. | | | | | |

| Supplementary Table 5: Cost analysis | | | |
| --- | --- | --- | --- |
|  | **Theatre** | **Clinic** | **Difference** |
| Expenses per patient |  |  |  |
| Consumables | -260 | -225 | +35 |
| Surgical Staff | -227 | -227 | 0 |
| Health Care Assistant | 0 | -9 | -9 |
| Theatre running costs including staff | -755 | 0 | +755 |
| Total Expenses per patient (£) | - 1,242 | - 461 | + 781 |

| Supplementary Table 6: PSQ selected questions results | | | |
| --- | --- | --- | --- |
| Questions | **Answers** | | |
|  |  | **Number (n)** | **%** |
| Did you have confidence and trust in the healthcare staff treating you? | Yes, always | 25 | 100 |
|  | Yes, sometimes | - | - |
|  | No | - | - |
| Were you given enough privacy when being examined or treated? | Yes, always | 25 | 100 |
|  | Yes, sometimes | - | - |
|  | No | - | - |
| Were you ever in any pain? | Yes | 1 | 4 |
|  | No | 24 | 96 |
| I would rather have had this procedure in the operating room? | Yes, definitely | - | - |
|  | Not sure | 2 | 8 |
|  | No | 23 | 92 |
| If I were to have this procedure again in the future, I would rather have a formal admission to hospital? | Yes, always | 1 | 4 |
|  | Yes, sometimes | - | - |
|  | No | 24 | 96 |
| Overall, did you feel you were treated with respect and dignity while you were in the hospital? | Yes, always | 25 | 100 |
|  | Yes, sometimes | - | - |
|  | No | - | - |
|  | | | |
|  | **Mean Score** | **SD** | **Range** |
| Overall Satisfaction | 9.68 | 0.85 | 6-10 |

# **Appendix 4 – Supplementary Figures**


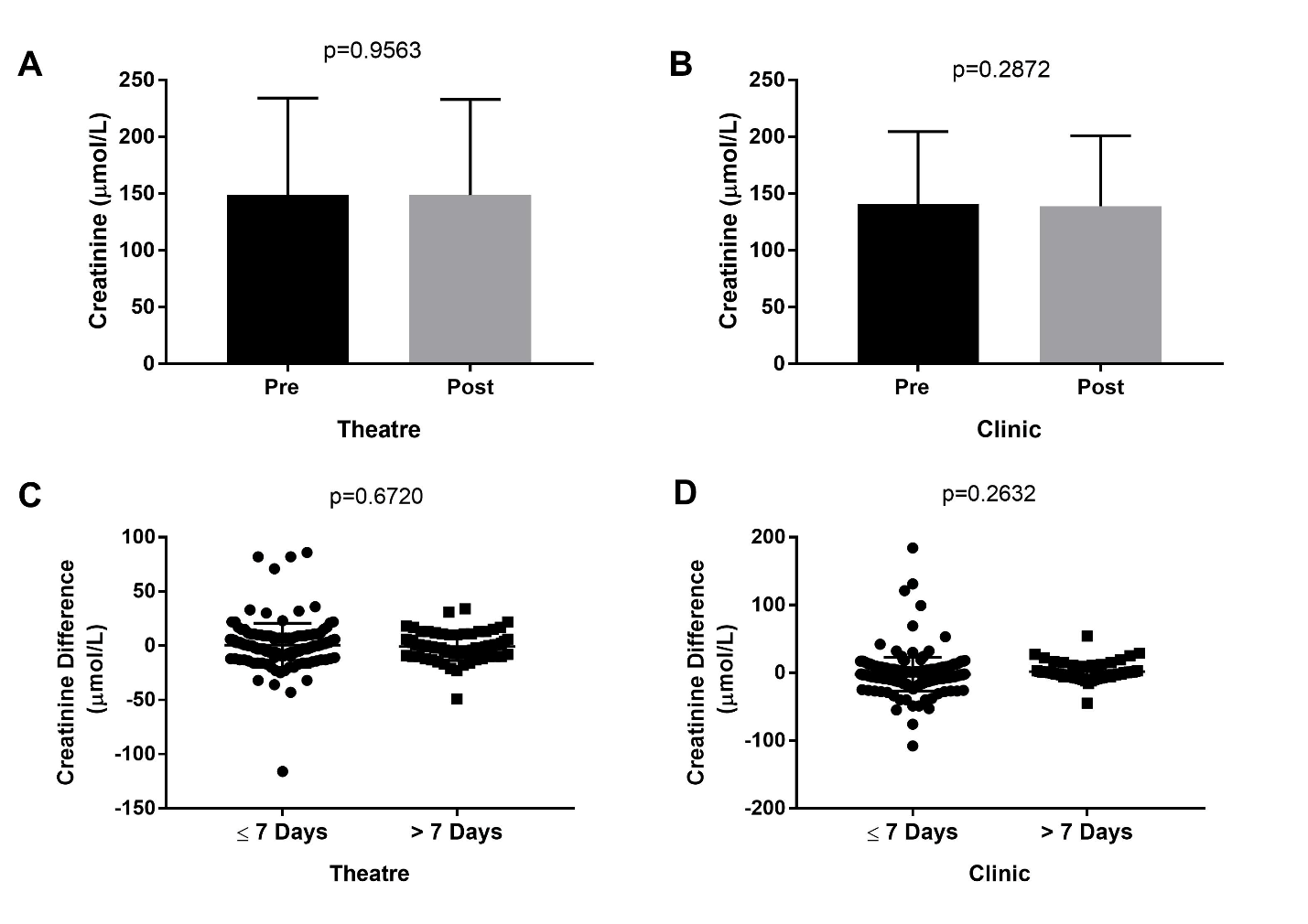


Supplementary Figure 1. Serum Creatinine levels were assessed pre- and post-removal of stents in clinic and in theatres, without any significant change in levels (paired t-test). Depending on how far along their transplant patients were, serum creatinine was checked at different intervals, with those having an early creatinine check ≤7 days between creatinine checks showing no significant difference to those whose serum creatinine was checked >7 days, post-removal of stent in both settings (unpaired t-test), when the Δ_Cr_ was compared.
